# A child that presents with an acute limp: Legg-Calvé-Perthes disease

**DOI:** 10.11604/pamj.2016.23.51.8871

**Published:** 2016-02-22

**Authors:** Kamran Khan

**Affiliations:** 1 Department of Orthopedic Surgery, Sinai Hospital of Baltimore,Baltimore, USA

**Keywords:** Avascular necrosis, Legg-Calvé-Perthes disease, perthes disease

## Image in medicine

A 6-year-old boy presented with the recent development of a right sided limp. He had no known medical conditions. On examination, the patient had limited internal/external rotation and abduction of the right hip. He walked with a non-antalgic gait and was noted to have a positive Trendelenberg sign. Lower extremity radiographs (A) revealed flattening of the right proximal femoral epiphysis with greater than 50% collapse. Mild acetabular changes of the right hip were present; however, there was still concentric reduction of the right femoral head within the acetabulum. After a thorough discussion of management options, the patient received botulinum toxin injections along the adductor longus and gracilis muscles to resolve an adduction contracture. An arthrogram (B) demonstrated mild-moderate flattening of the femoral head with lateral femoral head extrusion, and moderate femoral-acetabular congruency. The patient was subsequently given a hip abduction orthosis for intermittent daytime and full nighttime bracing, and was instructed to continue stretching exercises daily. The parents were educated regarding the cyclical nature of the pathology, and that the prognosis is relatively improved in younger children with the potential to still remodel. Legg-Calvé-Perthes disease describesan idiopathic avascular necrosis of the proximal femoral epiphysis. Initial therapy includes maintenance of the femoral head within the acetabulum by an abduction splint. Additionally, daily abduction stretching exercises and physical therapy are recommended. Surgical containment can be achieved by a femoral osteotomy to redirect the involved portion within the acetabulum.

**Figure 1:**
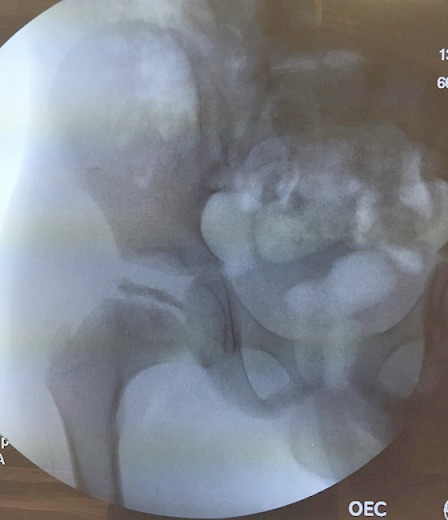
Arthrogram of the right hip

